# Molecular Mechanism of Action of Cycloxaprid, An Oxabridged *cis*-Nitromethylene Neonicotinoid

**DOI:** 10.3390/ijms24087511

**Published:** 2023-04-19

**Authors:** Yixi Zhang, Xiaoyong Xu, Jingting Wang, Xusheng Shao, Zewen Liu, Zhong Li

**Affiliations:** 1 Key Laboratory of Integrated Management of Crop Diseases and Pests (Ministry of Education), College of Plant Protection, Nanjing Agricultural University, 1 Weigang, Nanjing 210095, China; zhangyixi@njau.edu.cn (Y.Z.); 2022102106@stu.njau.edu.cn (J.W.); 2 Shanghai Key Laboratory of Chemical Biology, School of Pharmacy, East China University of Science and Technology, 130 Meilong Road, Shanghai 200237, China; xyxu@ecust.edu.cn (X.X.); shaoxusheng@ecust.edu.cn (X.S.); lizhong@ecust.edu.cn (Z.L.)

**Keywords:** cycloxaprid, nicotinic acetylcholine receptors, cockroach neurons, partial agonist

## Abstract

Cycloxaprid, an oxabridged *cis*-nitromethylene neonicotinoid, showed high insecticidal activity in Hemipteran insect pests. In this study, the action of cycloxaprid was characterized by recombinant receptor Nlα1/rβ2 and cockroach neurons. On Nlα1/β2 in *Xenopus* oocytes, cycloxaprid acted as a full agonist. The imidacloprid resistance-associated mutation Y151S reduced the *I*_max_ of cycloxaprid by 37.0% and increased *EC*_50_ values by 1.9-fold, while the *I*_max_ of imidacloprid was reduced by 72.0%, and *EC*_50_ values increased by 2.3-fold. On cockroach neurons, the maximum currents elicited by cycloxaprid were only 55% of that of acetylcholine, a full agonist, but with close *EC*_50_ values of that of *trans*-neonicotinoids. In addition, cycloxaprid inhibited acetylcholine-evoked currents on insect neurons in a concentration-dependent manner when co-applied with acetylcholine. Cycloxaprid at low concentrations significantly inhibited the activation of nAChRs by acetylcholine, and its inhibition potency at 1 µM was higher than its activation potency on insect neurons. Two action potencies, activation, and inhibition, by cycloxaprid on insect neurons provided an explanation for its high toxicity to insect pests. In summary, as a *cis*-nitromethylene neonicotinoid, cycloxaprid showed high potency on both recombinant nAChR Nlα1/β2 and cockroach neurons, which guaranteed its high control effects on a variety of insect pests.

## 1. Introduction

Nicotinic acetylcholine (ACh) receptors (nAChRs) are ligand-gated ion channels mediating fast cholinergic synaptic transmission in insect and vertebrate nervous systems [1,2]. The great abundance of nAChRs within the insect central nervous system (CNS) has led to the development of economically important insecticides targeting these receptors [3], of particular significance, the introduction of neonicotinoid insecticides such as imidacloprid in the early 1990s. Six other neonicotinoid compounds have been proven for use as insecticides, including nitenpyram (in 1995), acetamiprid (in 1996), thiamethoxam (in 1998), thiacloprid (in 2000), clothianidin (in 2002), and dinotefuran (in 2002). Neonicotinoid insecticides showed high toxicity and effective control of a range of insect pests, especially for Hemipteran insects with piercing-sucking feeding habitual nature. However, the intensive use of neonicotinoid insecticides in pest control has inevitably led to resistance in many insect pests [4,5]. Target insensitivity was an important mechanism for neonicotinoid resistance in insect pests, such as the brown planthopper *Nilaparvata lugens* and the green peach aphid *Myzus persicae*, although it may not be the prevalent mechanism in fields [6,7,8,9,10].

These neonicotinoid insecticides possess either an electron-withdrawing nitro (-NO_2_) or a cyano (-CN) group in *trans*-configuration, which has been postulated to contribute directly to their selectivity and high toxicity [3,11]. Cycloxaprid, an oxabridged *cis*-nitromethylene neonicotinoid with nitro (-NO_2_) group in *cis*-configuration, was designed and synthesized by researchers from the East China University of Science and Technology [12]. Cycloxaprid showed two distinct properties from imidacloprid and other commercial neonicotinoid insecticides. Cycloxaprid not only showed high toxicity to Hemipteran insect pests with piercing-sucking feeding, such as the cowpea aphid (*Aphis craccivora*), cotton aphid (*Aphis gossypii*), whitefly (*Bemisia tabaci*) and brown planthopper (*Nilaparvata lugens*), but also was seldom affected by the resistance to neonicotinoids in insect pests, such as imidacloprid resistance in *N. lugens*, *A. gossypii* and *B. tabaci* [13,14,15].

Cycloxaprid acted on insect nAChRs as the *trans*-configuration neonicotinoid insecticides, but it only had partially overlapped binding sites in the insect central nervous system [13]. However, its pharmacological properties on recombinant nAChRs are not systematically characterized. It is also unknown whether the pharmacological property of cycloxaprid in insect neuron cells will provide direct information to characterize the mode of action of cycloxaprid on insects. In this study, the action of cycloxaprid was studied on recombinant nAChRs composed of *N. lugens* Nlα1 subunit and *Rattus norvegicus* β2 subunit (Nlα1/β2) in *Xenopus* oocytes and native nAChRs in neuron cells isolated from *Periplaneta americana*. An imidacloprid resistance-associated mutation Y151S was introduced into *N. lugens* Nlα1 subunit, and its influence on cycloxaprid potency was evaluated on Nlα1^Y151S^/β2 nAChRs [8].

## 2. Results

### 2.1. Mode of Action of Cycloxaprid on Insect nAChRs

**Figure 1:**
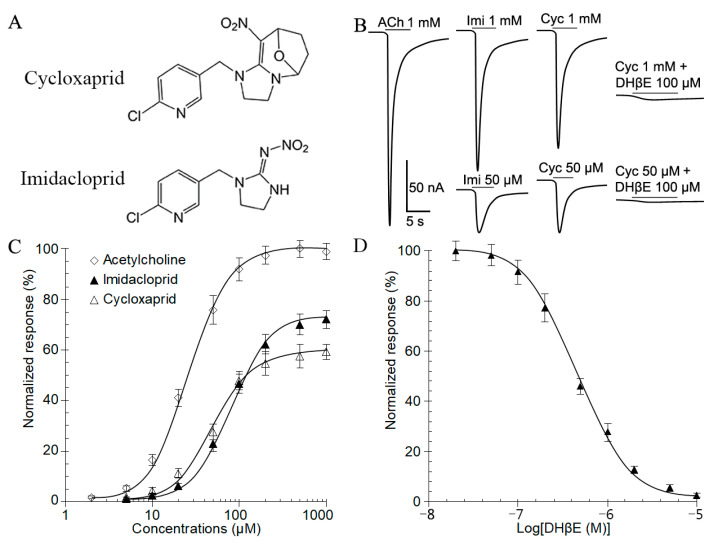
Cycloxaprid acted on recombinant receptor Nlα1/β2 in *Xenopus* oocytes as a full agonist. (**A**) Chemical structures of cycloxaprid and imidacloprid. (**B**) Representative currents elicited by acetylcholine, imidacloprid, and cycloxaprid on recombinant receptor Nlα1/β2 expressed in *Xenopus* oocytes. The concentrations (µM) were provided above the currents following drug name, acetylcholine (ACh), imidacloprid (Imi), and cycloxaprid (Cyc). (**C**) Concentration-response curve for inward currents induced by acetylcholine (*n* = 7), imidacloprid (*n* = 6), and cycloxaprid (*n* = 12). The currents were normalized to the maximum currents elicited by 1000 µM acetylcholine and presented as mean ± SEM. (**D**) The dose-inhibition relationship for hybrid receptor Nlα1/β2 (*n* = 11). The curve was obtained by perfusing oocytes with increasing concentrations of the antagonist DHβE for 30 s prior to application of 1000 µM cycloxaprid in the continued presence of the antagonist for 10 s. The data were normalized to the response of each oocyte to 1000 µM cycloxaprid and presented as mean ± SEM.

*N. lugens* nAChR subunit Nlα1 was co-expressed with the rat β2 subunit in *Xenopus* oocytes and the functional hybrid nAChRs were detected with evoked currents by agonists (Figure 1B, 1 mM ACh). The representative inward currents elicited by cycloxaprid were comparable to that by imidacloprid at 1 mM and 50 µM, which could be blocked by the nAChR-specific antagonist dihydro-β-erythroidine (DHβE) with *IC*_50_ of 0.47 ± 0.06 μM (Figure 1B,D). Dose-response tests with oocytes expressing Nlα1/β2 revealed that the *I*_max_ values of cycloxaprid (154.52 ± 15.02 nA) and imidacloprid (186.26 ± 13.75 nA) were similar, but both significantly less than that of acetylcholine (262.19 ± 14.33 nA). However, the *EC*_50_ value of cycloxaprid (49.1 ± 4.1 µM) on Nlα1/β2 was less than that of imidacloprid (71.0 ± 5.2 µM), but higher than that of acetylcholine (27.4 ± 3.3 µM) (Figure 1C and Table 1). The Hill coefficients of all 3 agonists on Nlα1/β2 were close to 1.0 (Table 1).

### 2.2. Influence of Mutation Y151S in Nlα1 on Cycloxaprid Potency

**Figure 2:**
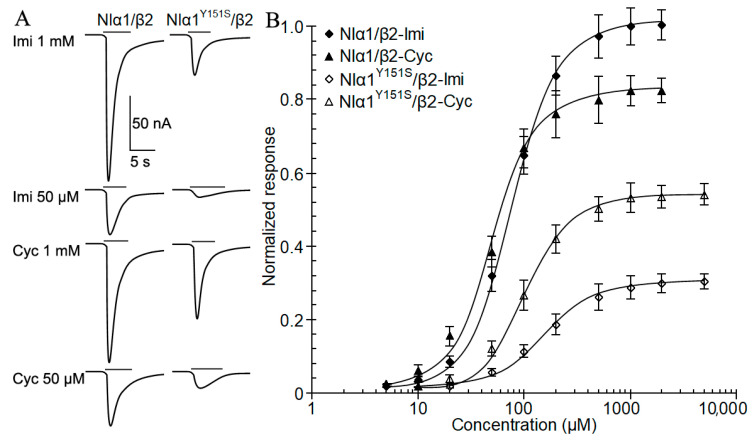
Effects of Y151S mutation on agonist potency on the receptor Nlα1^Y151S^/β2. (**A**) Representative currents elicited by imidacloprid and cycloxaprid on wildtype receptor Nlα1/β2 (left) and mutant receptor Nlα1^Y151S^/β2 (right). The concentrations (µM) were provided following drug name, imidacloprid (Imi) and cycloxaprid (Cyc). (**B**) Concentration-response curve for inward currents induced by imidacloprid and cycloxaprid (*n* = 6–12). The currents were normalized to the maximum currents elicited by 1000 µM imidacloprid on the wildtype receptor Nlα1/β2. Data were presented as mean ± SEM.

A previous study on an imidacloprid-resistant population of *N. lugens* identified a resistance-associated point mutation (Y151S) in nAChR subunit Nlα1 [8]. Here, we determined the influence of this mutation on imidacloprid and cycloxaprid potency on the recombinant receptors in *Xenopus* oocytes. The mutation decreased representative inward currents elicited by imidacloprid to 28.0% at 1 mM and 18.1% at 50 µM. In contrast, the mutation only decreased inward currents elicited by cycloxaprid to 63.0% at 1 mM and 31.4% at 50 µM (Figure 2A).

Comparisons of Nlα1/β2 and Nlα1^Y151S^/β2 receptors expressed in *Xenopus* oocytes revealed that Y151S mutation caused a significant rightward shift in the concentration-response curves of cycloxaprid and imidacloprid (Figure 2B). *EC*_50_ value of cycloxaprid was 49.12 ± 4.07 µM for Nlα1/β2 and 95.06 ± 7.18 µM for Nlα1^Y151S^/β2. This rightward shift of the cycloxaprid concentration-response curve (1.9-fold) caused by Y151S mutation was smaller than the shift of the imidacloprid curve (2.3-fold), which indicated Y151S mutation had less influence on cycloxaprid potency than that on imidacloprid. The Y151S mutation did not change the Hill coefficients of all 3 agonists, which were close to 1.0 (Table 1). In contrast, no significant differences in the *I*_max_, *EC*_50,_ and Hill coefficient of acetylcholine between Nlα1/β2 and Nlα1^Y151S^/β2, which were consistent with previous studies [9,16,17]. 

### 2.3. Mode of Action of Cycloxaprid on Cockroach DUM Neurons

**Figure 3:**
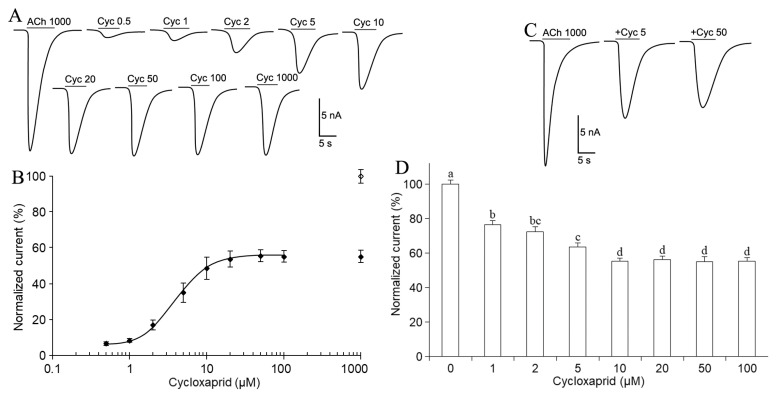
Cycloxaprid acted on cockroach DUM neurons as a partial agonist. (**A**) Representative currents elicited by acetylcholine and cycloxaprid on cockroach DUM neurons. The concentrations (µM) were provided above the currents following drug name, acetylcholine (ACh), and cycloxaprid (Cyc). (**B**) Concentration-response curve for inward currents induced by cycloxaprid (*n* = 12). The currents were normalized to the maximum currents elicited by 1000 µM ACh, as indicated by the blank diamond (◊). A separate filled diamond (♦) showed the normalized currents from 1000 µM Cyc. (**C**) Representative currents elicited by the application of ACh and co-application of ACh and Cyc on cockroach neurons (*n* = 15). Concentrations were provided following drug names. + meant ACh plus Cyc. (**D**) Cyc concentration-dependent inhibition on inward currents elicited by 1000 µM ACh on cockroach neurons (*n* = 14–20). The currents were normalized to the maximum currents elicited by 1000 µM ACh. Data were presented as mean ± SEM. We performed one-way ANOVA with Tukey’s multiple comparisons. Different letters indicated significant differences at 0.05 level.

On cockroach DUM neurons, inward currents (16.42 ± 2.26 nA, *n* = 16) were elicited by the application of 1 mM acetylcholine (Figure 3A). The inward current was characterized by a rapid rising phase to a peak amplitude followed by desensitization. The currents returned to zero steady-state level after washing the neurons with the drug-free saline. Cycloxaprid applications also elicited inward currents on cockroach neurons, which were dependent on cycloxaprid concentrations in a range of 0.5–20 µM, and the increase in cycloxaprid concentrations from 20 µM could not further amplify the currents (Figure 3A, *n* = 15–23). Cycloxaprid could not elicit a comparable current to that of acetylcholine, and the maximum currents elicited by cycloxaprid at concentrations of 20 μM–1 mM only reached 55% of that of 1 mM acetylcholine (Figure 3A, *n* = 17).

As mentioned above, cycloxaprid activated cockroach neuronal nAChRs in a concentration-dependent manner. Here, the concentration-response curve for inward currents induced by cycloxaprid was constructed. Fitted with the Hill equation, the calculated *EC*_50_ value for cycloxaprid on cockroach neurons was 3.82 ± 0.41 µM with the Hill coefficient of 1.07 ± 0.14 (Figure 3B, *n* = 12).

The co-application of 1 mM acetylcholine and cycloxaprid elicited smaller currents than that from the single application of 1 mM acetylcholine. The addition of 5 and 50 µM cycloxaprid reduced 36% and 45% of ACh-evoked currents on cockroach neurons, respectively (Figure 3C). Cycloxaprid at as low as 1 µM could significantly inhibit ACh-evoked currents when cycloxaprid was co-applied with 1 mM acetylcholine. The inhibition potency was dependent on the concentrations of cycloxaprid. The maximum inhibition was 45% by 10 µM cycloxaprid, and the cycloxaprid concentrations over 10 µM did not enhance the inhibition (Figure 3D). 

## 3. Discussion

### 3.1. Cycloxaprid Acted on Recombinant Receptor Nlα1/β2 as A Full Agonist

Cycloxaprid, the first *cis*-nitromethylene neonicotinoid insecticide, was developed by the East China University of Science and Technology in 2011 and registered in China in 2015 [18]. Cycloxaprid uniquely has the nitro group in the *cis*-configuration, whereas in all other commercial neonicotinoids, the nitro or cyano group is the *trans*-configuration [12]. Radioligand binding assay revealed that cycloxaprid affected the low-affinity binding site of imidacloprid in native *N. lugens* nAChRs, but with only partial overlap of imidacloprid binding sites [13]. To further understand the molecular mechanism of action of cycloxaprid, the pharmacological characteristics were evaluated and compared with that of imidacloprid in *Xenopus* oocytes expressing recombinant nAChRs Nlα1/β2 in the present study. The *EC*_50_ value of cycloxaprid on Nlα1/β2 was significantly less than that of imidacloprid, and the *I*_max_ elicited by cycloxaprid was similar to that of imidacloprid, which revealed that cycloxaprid had a higher agonist potency than imidacloprid, which was consistent with previous reports that cycloxaprid showed higher or comparable toxicities to imidacloprid against susceptible insect pests, such as *N. lugens*, *Aphis gossypii*, and *Bemisia tabaci* [13,14,15]. Furthermore, the inward currents elicited by cycloxaprid were concentration-dependent and could be completely blocked by the nAChR-specific antagonist DHβE. It illustrated that cycloxaprid was a selective and full agonist on nAChRs Nlα1/β2.

### 3.2. Cycloxaprid Showed Higher Agonist Potency on the Mutant nAChR Nlα1^Y151S^/β2

A point mutation Y151S was identified in nAChR subunit Nlα1 and Nlα3, which contributed to a high level of resistance in *N. lugens* to imidacloprid [8], and the mutation also significantly affected the potency of other *trans*-neonicotinoids except for dinotefuran [9]. This mutation was introduced into recombinant nAChRs Nlα1^Y151S^/β2 expressed in *Xenopus* oocytes and its influence on cycloxaprid potency was evaluated here. Compared to imidacloprid, cycloxaprid elicited higher inward current and showed a lower *EC*_50_ value on *Xenopus* oocytes expressing Nlα1^Y151S^/β2, which demonstrated that cycloxaprid had higher agonist potency than imidacloprid on the mutant nAChRs. Additionally, the mutation caused a less reduction in *I*_max_ value and an increase in the *EC*_50_ value of cycloxaprid than that of imidacloprid on recombinant receptors. The results indicated that the mutation Y151S had less influence on cycloxaprid potency than that of imidacloprid.

Although the metabolic mechanisms contributed by enhanced detoxification from P450s were prevalent for the resistance to imidacloprid and other *trans*-neonicotinoids in field populations of *N. lugens* [4,19,20], the Y151S mutation has been the first reported target insensitivity mechanisms for neonicotinoid resistance in insect pests [8,9]. The finding of Y151S mutation not only gave the first report of target insensitivity in insects but also provided information to identify native nAChRs targeted by neonicotinoids in insects [21]. The distinct structure property of *cis*-configuration neonicotinoid insecticides and compounds contributed importantly to the less effect of Y151S mutation and detoxification by neonicotinoid resistance-associated P450s, which also provided a rational explanation for the high toxicity of cycloxaprid to insect pests resistant to *trans*-neonicotinoids [13,14,15]. Cycloxaprid, as a novel neonicotinoid insecticide, possesses a *cis*-configuration nitro group, whereas other commercial neonicotinoids have either a nitro or a cyano group in *trans*-configuration [2,11,12,18]. In previous research, a *cis*-neonicotinoid analog IPPA152201 was reported to have excellent insecticidal activity against both susceptible strains and imidacloprid-resistant strains of *N. lugens*. Compared to the wildtype Nlα1/β2, this mutation reduced *I*_max_ for IPPA152201 to 63.2% and caused a 1.5-fold increase in *EC*_50_, which is much smaller than the effects on imidacloprid [22].

### 3.3. Cycloxaprid Acted on Cockroach Neuron as A Partial Agonist

On insect nAChRs, a partial agonist has two properties: First, it evokes much fewer currents than a full agonist such as acetylcholine. Second, it hinders receptor activation of other agonists when co-applied [23]. In this study, we found that the maximum currents elicited by cycloxaprid were much less than that of acetylcholine (1 mM), such as 55% on cockroach neurons. When cycloxaprid was co-applied with acetylcholine, the evoked currents were significantly less than that of acetylcholine alone at the same concentration. These data revealed that cycloxaprid acted as a partial agonist on cockroach neurons. It has been documented recently that neonicotinoid insecticide thiacloprid acted on cockroach Pameα7 homomeric nAChR as a partial agonist. Thiacloprid induced low inward currents, but the co-application or 5 min pretreatment with 10 µM thiacloprid decreased the nicotine-evoked current amplitudes by 54% and 28%, respectively [24].

The insect nAChR gene family consists of about 10 subunits, which can form various nAChRs including heteropentamers and homopentamers [1,2]. Diverse nAChRs, differing in subunit composition, have different electrophysiological and pharmacological profiles. For example, cockroach DUM neuron possesses α-bungarotoxin (α-Bgt) sensitive and insensitive receptors [25,26]. Two imidacloprid binding sites were observed in Hemipteran insects such as the aphid *Myzus persicae*, the leafhopper *Nephotettix cincticeps,* and the planthopper *N. lugens* [11,21,27]. As a result, the effect of a neonicotinoid insecticide on insect neurons revealed its comprehensive effect on multiple types of nAChRs, which would explain why cycloxaprid acted as a partial agonist on cockroach neurons.

As a partial agonist on nAChRs of insect neurons, cycloxaprid had two actions: eliciting inward current by itself and inhibiting currents of a full agonist. What action is more important for its modulation of insect nAChRs and consequently exposes its toxicity to insects? On honeybee Kenyon cells, imidacloprid at the low concentration of 10 µM could block 64% of the peak current amplitude evoked by 100 µM acetylcholine, although the currents evoked by 10 µM imidacloprid were less than 10% of the peak current [23]. Although the nAChRs of honeybee Kenyon cells (*EC*_50_ = 25.1 µM) were obviously less sensitive to imidacloprid than the receptors of other insect species, such as that on cockroach neurons (*EC*_50_ = 2.34 µM), imidacloprid had high toxicity to honeybees. Inhibition of acetylcholine-evoked currents by imidacloprid at low concentrations on honeybee Kenyon cells might provide a potential explanation for its high toxicity to honeybees. In the present study, at the low concentration of 1 µM singly applied or co-applied with acetylcholine, cycloxaprid only evoked the normalized currents of 8.5% on cockroach neurons, but it could inhibit the normalized currents of 23.4% from the full agonist acetylcholine. From these data, it seemed that the inhibition potency of cycloxaprid at 1 µM was higher than its activation potency on insect neurons. High toxicities of cycloxaprid to insect pests might also be from its inhibition of acetylcholine-evoked currents on insect neurons.

In summary, as an oxabridged *cis*-nitromethylene neonicotinoid, cycloxaprid possessed high and distinct potency on both recombinant Nlα1/β2 and cockroach neurons, as a full agonist and partial agonist, respectively. The selective activity on recombinant Nlα1/β2 with high potency and the complete blockage of nAChR-specific antagonist DHβE revealed that cycloxaprid acted on nAChRs. The distinct structural property and the weak impact of the Y151S mutation made cycloxaprid an excellent control agent for piercing-sucking insect pests and an alternative for *trans*-neonicotinoids to control insect pests that are resistant to current neonicotinoids. The distinct inhibition of the agonist-activated currents on insect neurons at a relatively low concentration of cycloxaprid provided another explanation for its high toxicity to a range of insect pests.

## 4. Materials and Methods

### 4.1. Chemicals

Acetylcholine (ACh), imidacloprid, and dihydro-β-erythroidine (DHβE) were purchased from Sigma-Aldrich (St. Louis, MO, USA). Cycloxaprid was synthesized and purified, as previously reported [12]. In the electrophysiological experiments, chemical solutions were freshly prepared in the modified Ringer’s solution (NaCl 150 mM, KCl 2.8 mM, HEPES 10 mM, MgCl_2_ 2 mM, atropine sulfate 0.5 µM; pH 7.2, adjusted with NaOH).

### 4.2. DUM Neuron Preparation

The DUM neuron cells were isolated from the sixth abdominal (A6) ganglion of *P. americana* male adults that were purchased from the Feitian Medicinal Animal Co., Ltd. (Danyang, Jiangsu, China) [25]. The ganglia were removed, unsheathed with forceps, and treated with collagenase (type I, 1 mg/mL) and Trypsin (1 mg/mL) in dissection saline (150 mM NaCl, 3 mM KCl, 10 mM HEPES, 10 mM glucose, pH 7.2) for 30 min at 37 °C. Then, ganglions were washed three times with the dissection saline supplemented with 5 mM CaCl_2_ and mechanically dissociated by repetitive gentle suctions through a Pasteur pipette. The neuron cell suspension was filtrated into the saline containing 5 mM of CaCl_2_, 10% fetal calf serum, 50 IU/mL of penicillin, and 50 μg/mL of streptomycin, then filtered through the 100 μM mesh sieve strainer, and then incubated at 37 °C. All operations were conducted under sterile conditions at 25 °C.

### 4.3. Electrophysiological Recording on DUM Neurons

Membrane currents on cockroach DUM neuron cells were recorded using the single electrode voltage clamp recording method at room temperature [25]. The microelectrode was fabricated from Clark borosilicate glass GC150TF (Warner Instruments, Hamden, CT, USA). Large neurons with a diameter of 50–100 mm were selected and impaled with 3M KCl-filled microelectrodes of 15–25 MΩ resistance. In a recording chamber (2 cm × 4 cm), the dissociated neurons superfused with the dissection saline supplemented with 5 mM CaCl_2_ at a flow rate of 0.5 mL/min. An amplifier (Multiclamp 700B Amplifier, Axon Instruments, Foster, CA, USA) was used to record membrane currents in a single electrode voltage clamp mode. Neurons were voltage-clamped at zero current potential (−40 to −95 mV). The electrodes were optimally compensated, and the switching rate was adjusted to 5–6 kHz. Recording conditions were optimized by adjusting capacitance neutralization. These data were recorded and analyzed by pClamp10 software (Axon Instruments, Foster City, CA, USA).

Drugs were applied by means of pressure ejection through a glass micropipette (Miniframe, Medical System Corporation, Indianapolis, IN, USA). The solution flowed at a constant rate of 0.5 mL/min from the opening of a 500 µm internal diameter Teflon tube placed 200 µm from the cell with pressure ejection of 5 psi. The drug pipette, with an opening of 5 µm, was aimed directly at the neuron from a distance of approximately 200 µm, perpendicular to the flow of the external solution. Thus, the drug solution could be washed away from the neuron with fresh external solution soon after the termination of ejection. Chemicals were dissolved and diluted in DMSO and diluted in the dissection saline, giving a final DMSO concentration <0.1%. In all experiments, 1 mM atropine was included in the external solution in order to block muscarinic receptors [25].

### 4.4. Expression of Hybrid nAChRs in Xenopus Oocytes

*N. lugens* nAChR Nlα1 subunit, its mutant Nlα1^Y151S^, and *Rattus norvegicus* β2 subunit were subcloned into the expression vector pGH19 as described previously [9]. Subunit cRNAs were generated using the mMESSAGE mMACHINE T7 transcription kit (ABI-Ambion, Foster, CA, USA).

*Xenopus* oocyte preparation and cRNA injection were performed as described previously [9]. Ovarian lobes were isolated from female *Xenopus laevis* according to standard procedures [28]. Clumps of stage V-VI oocytes were dissected in a sterile modified Barth’s solution (NaCl 88 mM; KCl 1 mM; MgCl_2_ 0.82 mM; CaCl_2_ 0.77 mM; NaHCO_3_ 2.4 mM; Tris-HCl 15 mM; with 50 U/mL penicillin and 50 μg/mL streptomycin; pH 7.4, adjusted with NaOH). The dissected oocytes were treated with collagenase (type IA, Sigma, St. Louis, MO, USA; 65 min at 18 °C, 245 units/mL in Barth’s solution, 10–12 oocytes/mL), rinsed and stored at 4 °C overnight, and manually defolliculated in the following day before injection with cRNA. The oocytes were incubated for approximately 60 h at 18 °C in Barth’s solution containing 5% heat-inactivated horse serum (Gibco/Invitrogen, Foster, CA, USA) and then stored at 4 °C. In all experiments, 50 ng (1 ng/nL) of each nAChR subunit was injected.

### 4.5. Electrophysiological Recording on Xenopus Oocytes

Electrophysiological recordings were made using a two-electrode voltage clamp (Multiclamp 700B Amplifier, Axon Instruments, Foster, CA, USA) as previously described [9]. Experiments were carried out at 18–20 °C between 2 and 6 days after injection. Oocytes, held in a 0.25 mL bath, were perfused at 4.5 mL/min with modified Ringer’s solution (NaCl 150 mM, KCl 2.8 mM, HEPES 10 mM, MgCl_2_ 2 mM, atropine sulfate 0.5 µM; pH 7.2, adjusted with NaOH) and voltage-clamped at −70 mV using the two-electrode clamp mode of a Multiclamp 700B Amplifier (Axon Instruments, Foster, CA, USA). The electrode resistance was 0.5–1 MΩ on the current-passing side. Experiments were terminated if the total holding current exceeded 2 μA in order to reduce the effect of series resistance errors.

The freshly prepared agonist solutions were applied via the bath perfusion for a period sufficient to obtain a stable plateau response (at low concentrations) or the beginning of a fall after a peak (at high concentrations). The inward current was recorded and digitized at 10 Hz for further analysis. An interval of 5 min was allowed between agonist applications, as this was found to be sufficient to ensure reproducible responses. In order to compensate for possible decreases in agonist sensitivity throughout the experiment, a standard concentration of agonist (approximately *EC*_20_ for the particular combination used) was applied every third response. The experiment was started only after checking that this standard concentration gave reproducible responses. The agonist application time was indicated in the bars above the response curves.

Antagonist dose-response relationships were obtained by perfusing oocytes with increasing concentrations of the antagonist for 30 s prior to the application of an agonist (1 mM) in the continued presence of the antagonist for 10 s.

### 4.6. Data Analysis

Dose-response curves for an agonist in electrophysiological recordings were fitted with the Hill equation to determine the maximum response (*I*_max_) and half-maximal activation concentration (*EC*_50_), as described previously [9]. In the Hill equation {*I* = *I*_max_/[1 + (*EC*_50_/x)^nH^]}, *I* is the response, *I*_max_ is the maximum response, *EC*_50_ is half-maximal activation concentration, x is the agonist concentration, and *n*H is Hill coefficient.

Dose-response curves for an antagonist in electrophysiological recordings were plotted against the concentrations of the antagonist on a logarithm scale and fitted with an equation, namely, *I* = 1/[1 + (x/IC_50_)*^n^*^H^], where *I* is the response, *IC*_50_ is the concentration to inhibit 50% of response of an agonist, x is antagonist concentration, and *n*H is Hill coefficient.

## Figures and Tables

**Table 1:** Agonist potency of acetylcholine, imidacloprid, and cycloxaprid on recombinant receptor Nlα1/β2 expressed in *Xenopus* oocytes.

Agonist	Nlα1/β2				Nlα1^Y151S^/β2			
	*I*_max_ (nA)	*EC*_50_ (µM)	Hill Coefficient	*n*	*I*_max_ (nA)	*EC*_50_ (µM)	Hill Coefficient	*n*
Acetylcholine	262.19 ± 14.33 a	27.36 ± 3.30 a	1.14	7	258.72 ± 18.53 a	30.08 ± 4.15 a	1.06	6
Imidacloprid	186.26 ± 13.75 b	71.01 ± 5.24 c	1.17	6	53.76 ± 8.92 c	162.25 ± 11.43 c	1.13	6
Cycloxaprid	154.52 ± 15.02 b	49.12 ± 4.07 b	1.08	12	98.82 ± 11.04 b	95.06 ± 7.18 b	1.15	11

One-way ANOVA with Tukey’s multiple comparisons was used to compare the *I*_max_ and *EC*_50_ of different chemicals on the recombinant receptors. Data in the table were mean ± SEM. The lowercase letters in the same column indicated the significant difference at 0.05 level.

## Data Availability

All data in this work are presented in this document.
